# Resistance of Food-Maintained Operant Responding to Mechanical Punishment in Rats: Further Evidence for Weak “Affective/Motivational Pain” in Rat Models of Inflammatory and Neuropathic Pain

**DOI:** 10.3389/fphar.2020.615782

**Published:** 2021-01-29

**Authors:** S. Stevens Negus, S. A. Marsh, E. A. Townsend

**Affiliations:** Department of Pharmacology and Toxicology, Virginia Commonwealth University, Richmond, VA, United States

**Keywords:** complete freund adjuvant, paclitaxel, Operant, punishment, naltrexone, U69,593, morphine

## Abstract

Clinically relevant chronic pain is often associated with functional impairment and behavioral depression as an “affective/motivational” sign of pain; however preclinical animal models of inflammatory and neuropathic pain often produce weak evidence of impaired function. We hypothesized that hindpaw mechanical stimulation produced by a requirement to rear on a textured “NOX” plate would punish operant responding in rats treated with intraplantar complete Freund’s adjuvant (CFA, a model of inflammatory pain) or the chemotherapeutic paclitaxel (PTX, a model of neuropathic pain) and produce sustained pain-related depression of operant behavior. Male Sprague–Dawley rats were trained under a progressive-ratio (PR) schedule of food-maintained operant responding, then treated with CFA (100 µL in left hindpaw), PTX (2.0 mg/kg IP on alternate days for four total injections; 6.6 mg/kg IV on alternate days for three total injections), or saline vehicle. PR break points and mechanical thresholds for paw withdrawal from von Frey filaments were then tracked for 28 days. Subsequently, rats were tested with the opioid receptor antagonist naltrexone to assess latent sensitization and with the kappa opioid receptor (KOR) agonist U69593 to assess KOR function. CFA produced significant mechanical hypersensitivity for 3 weeks but decreased PR breakpoints for only 1 day. Both IP and IV PTX produced mechanical hypersensitivity for at least three weeks; however, only IV PTX decreased PR breakpoints, and this decrease was not alleviated by morphine. After recovery, naltrexone reinstated mechanical hypersensitivity in CFA- but not PTX-treated rats, and it did not reinstate depression of breakpoints in any group. U69593 dose-dependently decreased PR breakpoints in all groups with no difference between control vs. CFA/PTX groups. These results suggest that rearing on a textured NOX plate was not sufficient to punish operant responding in CFA- and PTX-treated rats despite the presence of sustained mechanical hypersensitivity. The rapid recovery of operant responding could not be attributed to latent sensitization, KOR downregulation, or behavioral tolerance. These results extend the range of conditions under which putative chronic pain manipulations produce weak evidence for depression of operant responding as a sign of the “affective/motivational” component of pain in rats.

## Introduction

Clinically relevant chronic pain states in human and veterinary medicine are often associated with functional impairment, and restoration of function is a common goal of pain treatment (Dworkin et al., 2005; Jensen et al., 2007; Brown et al., 2008). Experimental models of inflammation and neuropathy have been developed in laboratory animals with the intent of inducing clinically relevant chronic pain states that include the so-called “affective/motivational” components of chronic pain; however, these models often produce surprisingly weak and transient evidence of impaired function (Negus, 2019; Tappe-Theodor et al., 2019; Gonzalez-Cano et al., 2020). For example, paclitaxel treatment to model chemotherapy-induced neuropathic pain in rats produced mechanical hypersensitivity of paw-withdrawal responses from von Frey filaments for at least four weeks, but it produced no significant changes in operant responding for electrical brain stimulation in an assay of intracranial self-stimulation (ICSS) and only weak and morphine-insensitive decreases in food-maintained operant responding (Legakis et al., 2018; Legakis et al., 2019). Similarly, spinal nerve ligation models of mononeuropathy that produced sustained mechanical hypersensitivity in rats for weeks failed to decrease either ICSS or food-maintained operant responding (Ewan and Martin, 2014; Okun et al., 2016).

It may be possible to enhance expression of functional impairment in operant procedures by supplementing the primary inflammatory or neuropathic insult with acute stimuli that are delivered as a consequence of behavior and that can function as punishers of behavior. As one example, Fuchs and colleagues developed a “place escape-avoidance procedure” (PEAP) in which inflammatory and neuropathic pain models were supplemented by acute, response-contingent mechanical stimulation to the affected paw (LaBuda and Fuchs, 2000a; LaBuda and Fuchs, 2000b). In this procedure, rats received intraplantar injection of complete Freund’s adjuvant (Ipl CFA, an inflammatory pain model) or unilateral L5 nerve ligation (a surgical mononeuropathy model) one or two days, respectively, before a sequence of two test days. On the first test day, mechanical paw withdrawal thresholds were assessed, and both Ipl CFA and L5 nerve ligation produced mechanical hypersensitivity in the injured paw. On the second day of testing, rats were placed into a chamber with equally sized light and dark compartments and a mesh floor that provided access to the subject’s feet from below. Locomotion in the normally preferred dark compartment resulted in delivery of an acute mechanical stimulus (476 mN von Frey filament) to the injured paw every 15 s, whereas locomotion in the light compartment resulted in delivery of the same stimulus to the uninjured paw every 15 s. Thus, delivery of the acute mechanical stimulus to the injured paw was contingent on locomotion in the dark compartment. This behaviorally contingent mechanical stimulation significantly reduced time in the dark compartment, indicating that mechanical stimulation of the injured paw functioned as a punisher of locomotion in the dark compartment, and a parallel study found that morphine blocked this punishment. This type of acute stimulus delivery is labor intensive, but Boada and colleagues recently developed a strategy to simplify mechanical stimulus delivery to the feet of rats with a nerve injury model of neuropathic pain (Boada et al., 2016). Specifically, they created a locomotor activity field divided into quadrants, and the floor of each quadrant consisted of a metal plate (called a “NOX plate”) covered with a grid of pyramids with increasing degrees of sharpness at their apex (apex areas of 1.5, 1.0, 0.6, 0.2 mm^2^). Mechanical stimulation (delivered via the rat’s body weight pressing its paw onto the pyramids) was contingent on locomotion in the different quadrants, and nerve-injured rats avoided the quadrant with the sharpest (0.2 mm^2^) pyramids, indicating that mechanical stimulation associated with these sharper pyramids punished locomotion in those quadrants.

The goal of the present study was to evaluate the degree to which acute mechanical stimulation delivered via NOX plates might function as a punisher of food-maintained operant responding and increase expression of behavioral depression in rats that had received Ipl CFA or repeated paclitaxel treatment. In addition to the NOX plates, several additional steps were taken in an effort to increase sensitivity of the procedure to pain-related behavioral depression relative to our previous study of chemotherapy effects on food-maintained operant responding (Legakis et al., 2019). First, only male rats were used, because only males showed evidence for paclitaxel-induced decreases in food-maintained operant responding in our previous study. Second, we used unsweetened grain pellets rather than sweetened pellets to reduce reinforcing effectiveness of the food reinforcer (Warner et al., 2015). Third, responding was maintained under a progressive-ratio (PR) schedule rather than a fixed-ratio schedule to permit determination of PR break points as a sensitive measure of CFA and paclitaxel effects on food reinforcing strength (Hodos, 1961; Richardson and Roberts, 1996). Fourth, the response lever was elevated to require rats to rear and place their full body weight on their hindpaws during operant responding, thus maximizing mechanical stimulation delivered via contact with the NOX plate. Lastly, we compared effects of our standard paclitaxel dosing regimen (4 alternate-day IP injections of 2.0 mg/kg/day paclitaxel; total dose = 8.0 mg/kg) with a more aggressive treatment regimen (3 alternate-day IV injections of 6.6 mg/kg/day; total dose = 19.8 mg/kg) shown to produce clinically relevant leukopenia as well as robust pain behaviors, including sensitivity to mechanical punishment in a PEAP apparatus (Hamity et al., 2017).

## Methods

### Subjects

Adult male Sprague-Dawley rats (Envigo, Somerset, NJ) that weighed 275–300 g upon arrival at the laboratory and were housed individually in an AAALAC International-accredited housing facility maintained on a 12-h light/dark cycle with lights on from 6:00 AM to 6:00 PM. All testing occurred during the light phase. Daily food rations (Teklad standard diet—19% protein; Envigo) were provided immediately after behavioral sessions and were titrated to maintain daily body weights within 5% of the running mean for each subject. In addition, rats had access to 45 mg food pellets (BioServ Dustless Precision Grain Pellets, #F0165, Flemington, NJ) during operant behavior sessions. Rats receiving IV paclitaxel received supplemental food as described below during paclitaxel treatment. Water was available ad libitum in the home cage. Animal-use protocols were approved by the Virginia Commonwealth University Institutional Animal Care and Use Committee and were in accordance with the National Academy of Science’s Guide for the Care and Use of Laboratory Animals (National Research Council, 2011).

### Drugs and Noxious Stimuli

Naltrexone HCl and U69593 (National Institute on Drug Abuse Drug Supply Program, Bethesda, MD) were dissolved in saline and administered subcutaneously (SC) in a volume of 1 ml/kg. Complete Freund’s adjuvant (CFA; Sigma Aldrich, St. Louis, MO) was administered to the intraplantar (Ipl) region of the left hindpaw in a volume of 100 µL. Controls received the same volume of Ipl saline.

Paclitaxel was obtained as a clinically available 6.0 mg/ml solution (Cardinal Health, Richmond, VA), and it was administered in one of two ways. One set of rats received a series of four intraperitoneal (IP) injections administered every other day. Each injection delivered 2.0 mg/kg paclitaxel (6.0 mg/ml stock solution × 0.33 ml/kg/day injection volume), and the total dose of 8.0 mg/kg was delivered over seven days as we have described previously (Legakis et al., 2018; Legakis et al., 2019). For a second set of rats, each rat was implanted with an intravenous (IV) catheter in the right jugular vein as described previously (Townsend et al., 2015; Townsend et al., 2019). Briefly, rats were anesthetized with isoflurane (2–3%), and the right jugular vein was isolated, punctured, and implanted with a custom-made polyethylene catheter. The unsecured end of the catheter was subcutaneously routed to the midscapular region of the back and connected to a subcutaneous vascular access port (Model VABR1B/22, Instech Laboratories, Plymouth Meeting, PA, United States). Subjects received one dose of ketoprofen (5 mg/kg) immediately following surgery and a second dose 24-h post-operatively. Catheters were flushed each weekday before and during paclitaxel injections with an antibiotic (gentamicin, 0.08 mg) followed by 0.1 ml of heparinized saline (30 U/ml). Beginning one week after surgery, rats received a series of three IV injections administered every other day. Each injection delivered 6.6 mg/kg paclitaxel, and the total dose of 19.8 mg/kg was delivered over 5 days as described previously (Hamity et al., 2017). For each day of IV treatment, the 6.0 mg/ml stock solution of paclitaxel was diluted in saline to a concentration of 0.66 mg/ml, and a 1.0 ml/kg injection was administered once every 10 min for a total of 10 injections (total daily injection volume of 10 ml/kg). Pilot studies with this IV paclitaxel dosing regimen indicated a risk of substantial weight loss; accordingly, during the week of IV paclitaxel treatment, rats received a supplemental diet of powdered grain and sucrose pellets mixed with peanut butter and DietGel®31 M (ClearH2O, Portland, ME) and placed on the cage floor for easy access. Paclitaxel controls received four IP injections of saline.

### Overview of Experimental Design

**FIGURE 1:**
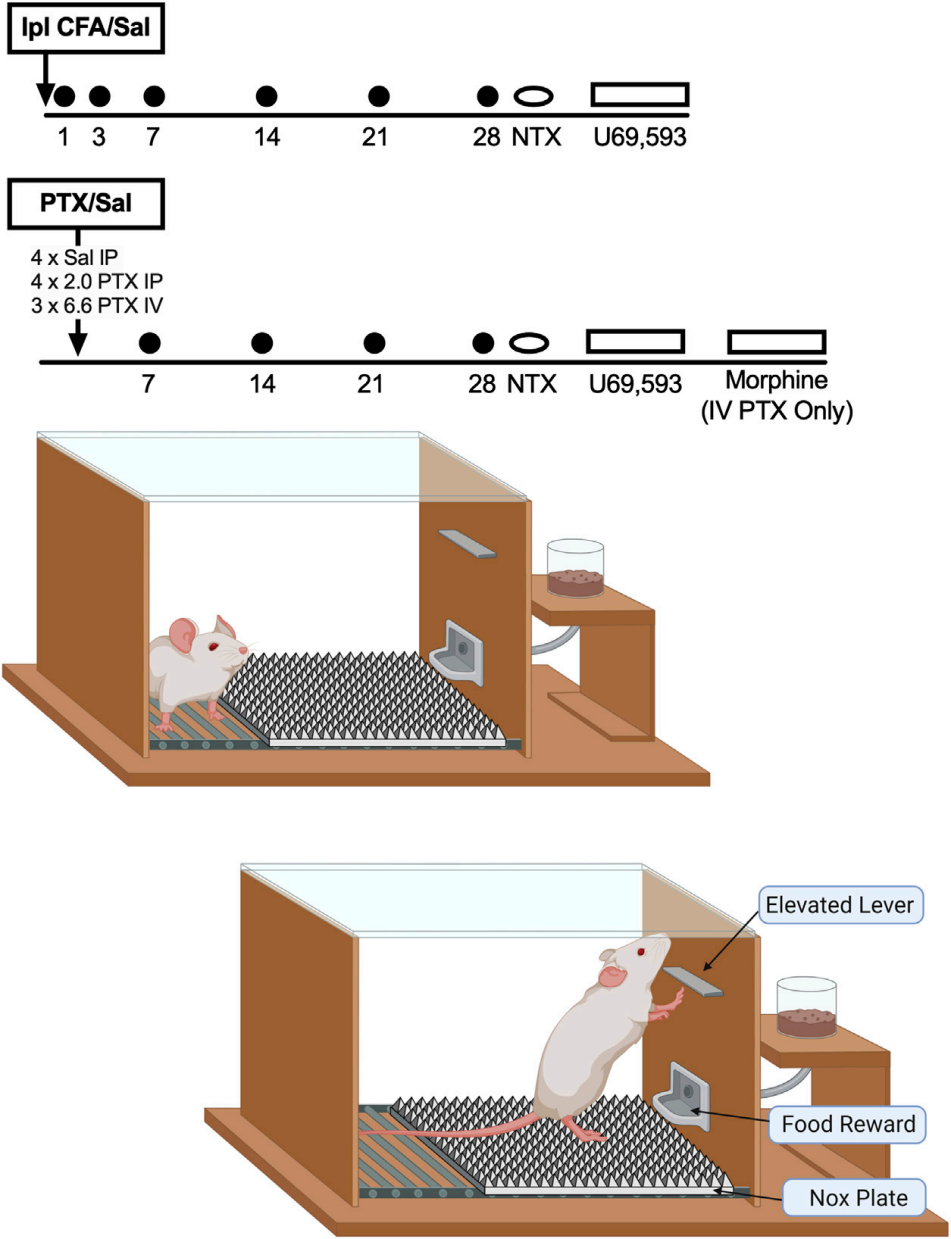
Experimental design and apparatus. Timelines at the top show the sequence of treatments and tests in each group. Operant responding was assessed each weekday, and mechanical sensitivity to von Frey filaments was assessed on days indicated by the filled circles. Schematics of the operant chamber and position of the NOX plate are shown at the bottom. Rats had to rear on the NOX plate to reach the lever and complete the response requirement.

The main goal of the study was to compare the time courses of mechanical hypersensitivity and mechanical punishment of operant responding in rats after treatment with CFA, paclitaxel, or their respective controls. Figure 1 shows the timeline of experimental events. After collection of baseline data, rats received their respective treatments, and both mechanical sensitivity and operant responding were assessed over a period of 28 days. Sessions of food-maintained operant responding were generally conducted during all weekdays, whereas mechanical sensitivity was assessed approximately 1 h after operant behavioral sessions and after delivery of supplemental food rations on the days indicated in Figure 1. After the conclusion of this 28-days test period, rats were treated with 3.2 mg/kg naltrexone 15 min before an operant behavioral session or before mechanical sensitivity testing on Days 29 and 31. The order of naltrexone testing with operant vs. mechanical sensitivity assessments was counterbalanced across rats. When naltrexone was tested before an operant session, mechanical sensitivity testing was omitted afterward. When naltrexone was tested before mechanical sensitivity assessments, rats had their usual operant session followed by naltrexone administration and then by mechanical sensitivity assessments. Effects of the opioid receptor antagonist naltrexone were evaluated to assess potential presence of latent sensitization (Corder et al., 2013). Next, on Days 35–38, each rat was tested with a range of U69593 doses (vehicle, 0.1, 0.32, and 1.0 mg/kg SC) administered 15 min before operant behavioral sessions. All rats received all doses on four consecutive days, and dose order was randomized across rats using a Latin Square design. Effects of the kappa opioid receptor (KOR) agonist U69593 were examined to assess potential changes in KOR agonist effects that might be indicative of pain-related changes in KOR signaling (Liu et al., 2019). A final study with the mu opioid receptor (MOR) agonist morphine was conducted in rats treated with high-dose IV paclitaxel. On Days 42–45 after initiation of IV paclitaxel, each rat was tested with a range of morphine doses (vehicle, 0.32, 1.0, 3.2 mg/kg SC) administered 30 min before operant behavioral sessions. As with U69593, all rats received all morphine doses on four consecutive days, and dose order was randomized across rats using a Latin Square design. Morphine was tested to evaluate effectiveness of a clinically effective analgesic to increase operant responding in IV paclitaxel-treated rats (the only group to show a sustained decrease in operant responding) and hence to evaluate the role of pain in mediating that decrease. Morphine was tested at doses shown previously to reverse paclitaxel-induced mechanical hypersensitivity (Legakis and Negus, 2018).

### Mechanical Sensitivity Testing With von Frey Filaments

On test days, rats were placed on an elevated mesh galvanized steel platform in individual chambers with a hinged lid and allowed to acclimate for at least 20 min before exposure to mechanical stimuli. Von Frey filaments (ranging from 0.4 to 15.0 g and increasing in ∼0.25 log increments; North Coast Medical, Morgan Hill, CA) were applied to the plantar surface of each hindpaw, and the threshold stimulus to elicit paw withdrawal was determined in log grams using the “up-down” method as previously described (Chaplan et al., 1994; Leitl et al., 2014b; Legakis et al., 2019). Filament forces greater than 15.0 g were not used because they physically lifted the paw, and as a result, paw movement could not be reliably attributed to a withdrawal response by the subject. For rats receiving Ipl CFA or saline, only data from the injected paw were subjected to data analysis; thresholds in the uninjected paw were nearly always at the cutoff value of 15.0 g (data not shown). For rats receiving paclitaxel or saline, data from both paws were averaged within a rat and then across rats for subsequent analysis.

### Food-Maintained Operant Responding in Food-Restricted Rats

#### Apparatus

Studies were conducted in sound-attenuating boxes containing modular acrylic and metal test chambers (Med Associates, St Albans, VT), and major features of the operant chambers are illustrated in Figure 1. Each chamber contained one active response lever, three stimulus lights (red, yellow, and green) centered above the lever, a 2-W house light, a floor made of parallel bars, and a pellet dispenser that delivered 45 mg grain pellets to an aperture beside the lever. Control of stimulus delivery in the operant chamber and collection of data on lever presses emitted and reinforcements earned were accomplished with a computer, interface, and custom software (Med PC-IV, Med Associates).

#### Training

Training progressed in three phases in two different sets of operant chambers that were identical except for the height of the response lever (6 vs. 15 cm above the floor in the low-lever and high-lever chambers, respectively). Phase 1 of training was conducted in the low-lever chambers. Operant responding was established for a series of increasing fixed-ratio (FR) values from FR 1 to FR 10, and the schedule of reinforcement was then changed to a progressive-ratio (PR) schedule such that the response increment for each reinforcer increased exponentially (1, 2, 4, 8, 16, etc.) with each set of eight reinforcers. Thus, the response requirement increased by an increment of 1 for each of the first eight reinforcers (1, 2, 3, 4, 5, 6, 7, 8), an increment of two for the second set of eight reinforcers (10, 12, 14, 16, 18, 20, 22, 24), an increment of four for the third set of eight reinforcers (28, 32, 36, 40, 44, 48, 52, 56), and so on. The break point was defined as the number of reinforcers earned during a session, and training on the PR schedule in the low-lever boxes was considered complete when the breakpoints over three consecutive days varied by ≤ 10% of the mean with no upward or downward trends. In Phase 2 of training, rats were moved to the high-lever boxes, which required rats to rear to reach the lever (see Figure 1), and training progressed again through the same sequence of schedules until responding again stabilized under the PR schedule. For the third and final phase of training, a textured aluminum “NOX” plate (Control Technologies, Hickory, NC) was inserted onto the floor of the high-lever chambers (see Figure 1). The 23.5 × 18.5 cm NOX plate spanned the width of the floor beneath the response lever and extended across approximately 2/3 of the cage floor toward the opposite wall. The remaining 10.5 cm adjacent to the opposite wall was not covered and provided a location where the rat could avoid the NOX plate. The NOX plate surface consisted of a grid of pyramids (5 mm^2^ at base and 5 mm tall), each with an apex of 0.2 mm designed to activate sensitized nociceptors in a partial spinal-nerve-ligation model of neuropathy (Boada et al., 2016). To reach the lever, each rat had to rear on the NOX plate and experience the mechanical stimulation associated with the force of its body weight pressing its hindpaws against the apices on the NOX plate. Training continued until responding again stabilized under the terminal PR schedule. With one exception (see below), all remaining sessions of food-maintained operant responding took place in the high-lever chambers with the NOX plate in place beneath the lever.

#### Testing

Once stable baseline break points had been established under the high-lever/NOX plate conditions, groups of *N* = 6 rats each were treated with 1) Ipl CFA (single injection), 2) Ipl saline (single injection), 3) IP paclitaxel (four doses of 2.0 mg/kg/day administered on alternate days), 4) IV paclitaxel (three doses of 6.6 mg/kg/day administered on alternate days), and 5) IP saline (four injections on alternate days). Operant behavioral testing then proceeded as shown in Figure 1. As described below, results suggested that depression of operant responding was more transient than mechanical hypersensitivity after both CFA and paclitaxel. To assess possible behavioral factors in this resilience of operant responding, two additional groups (*N* = 5 per group) were tested with CFA using a modification of the design shown in Figure 1. For the first group, operant behavioral sessions were suspended for seven days after CFA injection before resuming from Day 7–14. This manipulation was implemented to delay any learning of postural adjustments that might relieve pressure on the inflamed paw and thereby permit recovery of depressed operant responding. If learned postural adjustments contributed to rapid recovery of operant responding, then responding was expected to be lower on Day 7 in the group that did not have that opportunity for learning between Days 1 and 7. For the second group, rats were trained as described above, but then returned to the low-lever chambers and rebaselined with the low lever and bar floor (i.e. no NOX plate). CFA was then administered, and operant responding was assessed for 14 days. This manipulation was implemented to evaluate the degree to which mechanical stimulation produced by rearing on the NOX plate functioned as a punisher of operant responding. If rearing on the NOX plate functioned as a punisher, then operant responding would be lower in the high-lever/NOX plate condition where this stimulus was present than in the low-lever/bar-floor context where this stimulus was absent.

### Data Analysis

Baseline mechanical thresholds and operant break points were compared by one-way ANOVA. For analysis of CFA, paclitaxel, and control treatment effects on operant responding, break points were expressed as a percent of each rat’s baseline break point determined before CFA, paclitaxel, or control treatment. Mechanical thresholds and % baseline operant break points were evaluated by two-way ANOVA, with time as a within-subject variable and treatment as a between-subject variable. Similarly, naltrexone and U69593 effects were compared across groups with dose as a within-subject variable and treatment as a between-subject variable. Morphine was tested in only one group, and effects were analyzed by one-way ANOVA. A significant one-way ANOVA or a significant treatment × day interaction in a two-way ANOVA was followed by a Holm–Sidak post hoc test, and the criterion of significance for all tests was *p* < 0.05.

## Results

When data were collapsed across groups for each of the three different phases of operant training, there were no significant differences in break points under Phase 1 low-lever/bar-floor, Phase 2 high-lever/bar-floor, or Phase 3 high-lever/NOX plate contexts (mean ± SEM break points = 25.8 ± 0.8, 24.3 ± 0.5, and 24.9 ± 0.7 pellets per session, respectively). Additionally, there were no significant differences in break points across groups at the conclusion of operant training, and similarly, there were no differences across groups in baseline mechanical thresholds (overall mean ± SEM = 1.13 ± 0.02 log g). Rats in the IV paclitaxel group were surgically implanted with IV catheters and allowed to recover for one week prior to initiation of paclitaxel treatment, and mean ± SEM break points in this group did not differ at the conclusion of operant training (22.9 ± 1.5), on the day after surgery (22.0 ± 2.9), or after the one-week recovery period (24.9 ± 2.6).

**FIGURE 2:**
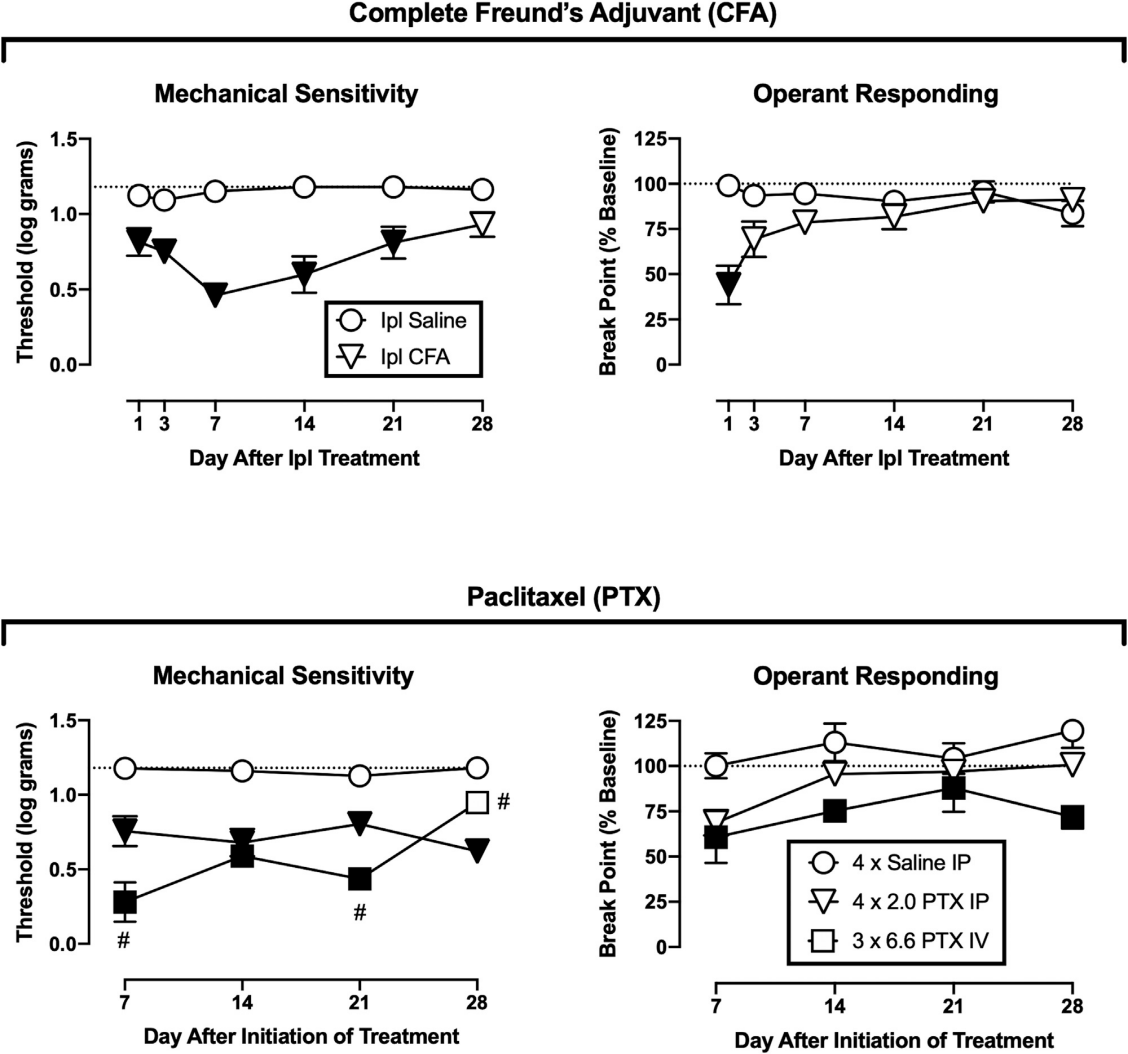
Time course of changes in mechanical sensitivity and operant responding after treatment with CFA **(top panels)** or paclitaxel **(bottom panels)**. Abscissae: days after CFA treatment or initiation of paclitaxel treatment. Ordinates: Threshold von Frey stimulus to elicit paw withdrawal (expressed as log g, **left panels**) or PR break point (expressed as a % of the pre-CFA/PTX baseline, **right panels**). All points show mean ± SEM from *N* = 6 rats except IV PTX (*N* = 5). Filled points indicate a significant difference from saline controls, and the hashtag (#) indicates a significant difference from IP PTX, *p* < 0.05.

Figure 2 shows the time course of changes in mechanical paw-withdrawal thresholds and operant break points after treatment with CFA or its vehicle (top panels) or paclitaxel or its vehicle (bottom panels). For Ipl CFA, decreases in operant break points were significant but more transient than decreases in mechanical thresholds. CFA significantly reduced mechanical thresholds from Days 1–21 [treatment × day interaction, F(5,50) = 4.59, *p* = 0.002], whereas operant breakpoints were reduced only on Day 1 [treatment × day interaction, F(5,50) = 10.110, *p* < 0.001].

For paclitaxel, one rat in the IV treatment group lost sufficient body weight to meet IACUC-established moribundity criteria. As a result, this rat was euthanized on Day 11 after initiation of paclitaxel treatment, its data are omitted from all data analysis, and the IV paclitaxel results show data only from the remaining five rats. Both the lower dose IP regimen and the higher dose IV regimen of paclitaxel treatment significantly reduced mechanical thresholds [treatment × day interaction, F(6,42) = , *p* < 0.001]. Relative to IP saline treatment, mechanical thresholds were reduced across all test days by IP paclitaxel and across Days 7–21 by IV paclitaxel. Additionally, IV paclitaxel produced greater mechanical hypersensitivity than IP paclitaxel on Days 7 and 21, but significantly less on Day 28. Paclitaxel also significantly reduced operant responding, and because there was a main effect of treatment [F(2,14) = 7.821, *p* = 0.005] but not a significant treatment × day interaction, analysis defaulted to a one-way ANOVA by treatment. Relative to IP saline treatment, operant responding was significantly reduced by IV paclitaxel. Differences between IP saline and IP paclitaxel (*p* = 0.089), and between IP and IV paclitaxel (also *p* = 0.089), approached but did not meet the criterion for significance. Thus, IP paclitaxel produced mechanical hypersensitivity without significantly altering operant responding, whereas IV paclitaxel produced lethality in one rat and both mechanical hypersensitivity and reduced operant responding in the remaining rats.

**FIGURE 3:**
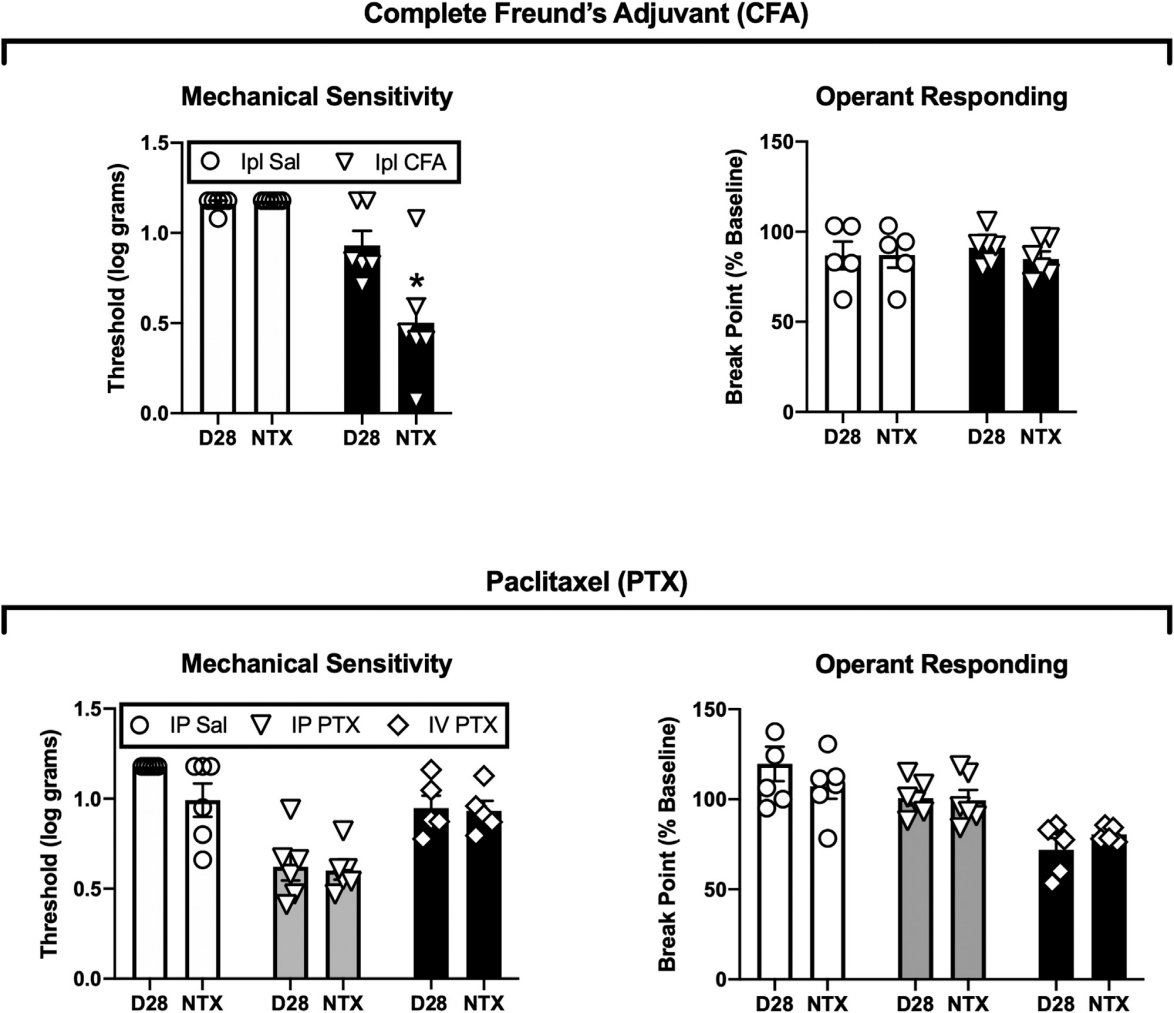
Effects of naltrexone on mechanical sensitivity and operant responding after treatment with CFA **(top panels)** or paclitaxel **(bottom panels)**. Abscissae: Data from Day 28 (D28) or the naltrexone-treatment day (NTX, either Day 29 or 31). Ordinates: Threshold von Frey stimulus to elicit paw withdrawal (expressed as log g, **left panels**) or PR break point (expressed as a % of the pre-CFA/PTX baseline, **right panels**). All bars show mean ± SEM from *N* = 6 rats except IV PTX (*N* = 5). The asterisk indicates a significant difference from D28, *p* < 0.05.

Figure 3 shows the effects 3.2 mg/kg naltrexone on mechanical thresholds and operant break points on Days 29 and 31 after Ipl CFA or initiation of paclitaxel treatment. In CFA-treated rats, naltrexone reinstated mechanical hypersensitivity [treatment × naltrexone interaction, F(1,10) = 15.52, *p* = 0.003], but it had no effect on operant break points, and it did not affect either endpoint in the saline-treated control rats. Naltrexone did not significantly alter either mechanical thresholds or operant break points in paclitaxel-treated rats or their saline-treated controls. In IP paclitaxel-treated rats, mechanical sensitivity recovered to baseline levels by 12 weeks after paclitaxel treatment, but naltrexone still failed to reinstate mechanical hypersensitivity or produce depression of operant responding at this time (data not shown).

**FIGURE 4:**
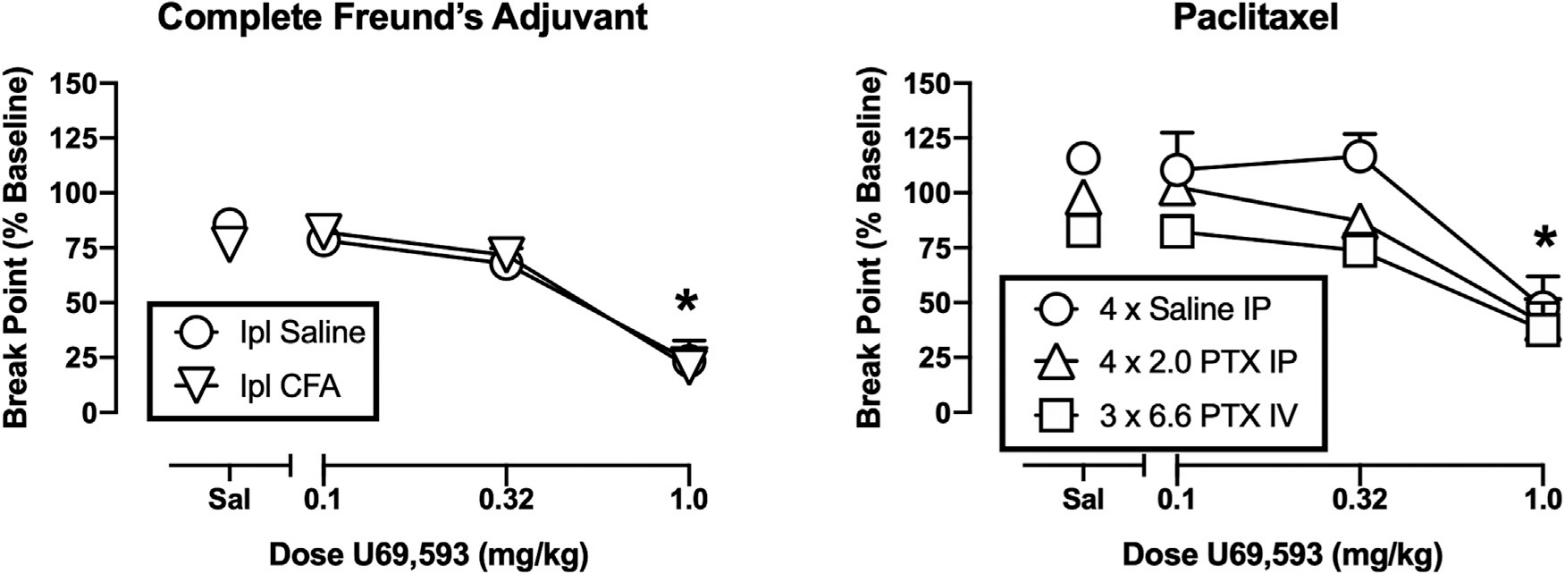
Effects of U69593 on operant responding after treatment with CFA (**left panel)** or paclitaxel **(right panel)**. Abscissae: Dose U69593 (mg/kg, log scale). Ordinates: PR break point expressed as a % of the pre-CFA/PTX baseline. All points show mean ± SEM from *N* = 6 rats except IV PTX (*N* = 5). The asterisk indicates a significant difference from saline (Sal) for all groups, *p* < 0.05.

Figure 4 shows the effects of U69593 on operant responding. The high dose of 1.0 mg/kg U69593 significantly decreased break points in both the CFA-treated rats and their saline-treated controls [main effect of U69593 dose, F(3,27) = 62.30, *p* < 0.001], but there was no difference between groups. Similarly, 1.0 mg/kg U69593 significantly decreased break points in both paclitaxel treatment groups and in the saline-treated controls [main effect of U69593 dose, F(3,39) = 59.13, *p* < 0.001]. In this study, the main effect of treatment approached but did not reach the criterion for significance [F(2,13) = 6.242, *p* = 0.072], and there was no treatment × U69593 interaction. Thus, while IV paclitaxel-treated rats tended to have lower breakpoints across all U59593 doses, there was no significant difference in U69593 effects across groups. Table 1 shows the effects of morphine determined 42–45 days after treatment with high-dose IV paclitaxel. Break points after morphine vehicle (i.e. saline) were still significantly lower than baseline (*t* = 2.403, *p* = 0.037); however, morphine did not alleviate this operant depression, and instead tended to reduce PR break points further.

**TABLE 1:** Morphine did not increase operant responding in rats treated with high-dose IV paclitaxel, and instead tended to decrease break points further.

Morphine dose (mg/kg)	% Baseline breakpoint (Mean ± SEM)
Saline	92.33 ± 2.53
0.32	91.74 ± 3.5
1.0	81.95 ± 4.78
3.2	64.36 ± 11.50

**FIGURE 5:**
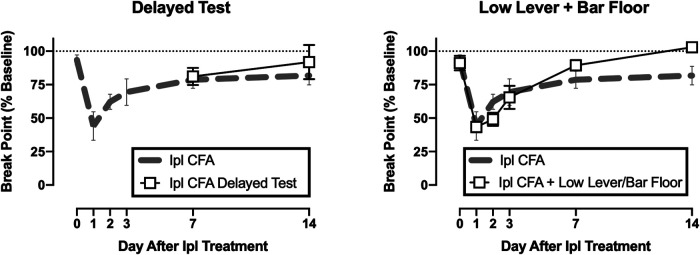
Effects of delayed operant testing **(left panel)** or operant testing with a low lever and bar floor **(right panel)** in CFA-treated rats. Abscissae: days after CFA treatment. Ordinates: PR break point expressed as a % of the pre-CFA baseline. All points show mean ± SEM from *N* = 5 rats. There were no differences between operant break points in rats tested under these altered parameters (squares) in comparison to data collected under the original parameters (gray dashed line, from Figure 2).

Figure 5 shows CFA effects on operant responding under two additional conditions. First, suspension of access to operant responding did not delay recovery of operant responding. Break points on Days 7 and 14 after CFA were similar regardless of whether rats engaged in operant responding on Days 1–6 or not. Second, the time course of CFA-induced decreases in operant break points was similar regardless of whether rats were tested in the high-lever/NOX-plate context or the low-lever/bar-floor context. There was a significant interaction between test context and time [F(5,45) = 2.746, *p* = 0.030]; however, post hoc analysis did not reveal a difference between groups on any day.

## Discussion

This study examined the degree to which mechanical stimulation to the hindpaws might elicit the “affective/motivational” component of chronic pain as indicated by pain-related punishment of operant responding maintained under a PR schedule of food delivery in rats treated with either Ipl CFA (to produce paw inflammation) or repeated paclitaxel (to produce polyneuropathy). Mechanical stimulation to the hindpaws was achieved by requiring rats to rear on a textured metal “NOX” plate to reach the operant response lever. There were three main findings. First, the requirement to rear on the NOX plate did not function as a punisher in the absence of putative pain manipulations. PR break points during the three phases of initial training were identical regardless of whether rats had to rear on the NOX plate or not. Second, both CFA and paclitaxel produced significant depression of operant responding when rats were required to rear on the NOX plate; however, as discussed further below, these decreases in operant responding did not appear to reflect pain-related punishment. Lastly, studies with naltrexone, U69593, and delayed operant access suggested that rapid recovery of operant responding did not reflect processes of latent sensitization, altered KOR signaling, or learned compensatory behaviors. Overall, these results extend the range of conditions under which operant responding in rats is resistant to behavioral depression by putative chronic pain treatments.

Clinically relevant chronic pain states are often accompanied by functional impairment and decreases in activities of daily living, and a major goal of pain treatment is to restore normal function. Insofar as one goal of preclinical research is to model clinically relevant pain behaviors and predict effectiveness of candidate treatments, one theme of preclinical research has been to evaluate the degree to which acute, inflammatory, and neuropathic pain models produce analgesic-reversible decreases in unconditioned or operant conditioned behaviors in laboratory animals as a measure of the “affective/motivational” component of pain (Negus, 2019; Tappe-Theodor et al., 2019; Gonzalez-Cano et al., 2020). As one example, intraperitoneal injection of dilute acid (IP acid) can serve as an acute chemical noxious stimulus in rats and mice to decrease a range of unconditioned behaviors (e.g. nesting, locomotion, wheel running, consumption of palatable food) and operant behaviors (e.g. lever pressing for food or electrical brain stimulation) (Stevenson et al., 2006; Stevenson et al., 2009; Miller et al., 2011; Negus, 2013; Negus et al., 2015; Cone et al., 2018). Moreover, in many cases, these IP acid-induced decreases in behavior can be blocked by clinically effective analgesics (e.g. nonsteroidal anti-inflammatory drugs, MOR agonists) but not by many types of nonanalgesics (e.g. KOR agonists, cannabinoid 1 receptor agonists, neurokinin receptor antagonists) that often produce false-positive effects in conventional assays of reflexive pain behaviors (Negus, 2019). These types of results have been interpreted to suggest that preclinical assays of pain-related behavioral depression could have both face validity as models of clinically relevant functional impairment and predictive validity for evaluation of candidate analgesics.

Pain-related and analgesic-reversible behavioral depression has also been produced in laboratory animals by other noxious stimuli, but in many cases, behavioral depression is weak or transient in comparison to the time course of more commonly used pain behaviors in preclinical pain research. In particular, many procedures have been developed to model chronic inflammatory and neuropathic pain states in laboratory animals, and the chronicity of the pain state is typically claimed on the basis of sustained hypersensitivity of withdrawal reflexes to mechanical or thermal stimuli (e.g. sustained decreases in mechanical thresholds for paw withdrawal from von Frey filaments) (Le Bars et al., 2010). For example, Ipl CFA to model inflammatory pain routinely produces mechanical hypersensitivity for days to weeks, whereas chemotherapy treatments to model chemotherapy-induced neuropathic pain produce mechanical hypersensitivity for weeks to months (Stein et al., 1988; Polomano et al., 2001; Leitl et al., 2014b; Legakis et al., 2018). However, in contrast to the sustained functional impairment that drives many chronic pain patients to seek medical care, Ipl CFA and chemotherapy treatments typically reduce operant conditioned behaviors for only a few days if at all (Leitl et al., 2014b; Legakis et al., 2018; Legakis et al., 2019). This transience of behavioral depression reduces both face validity of the preclinical procedures and utility of these procedures for evaluating chronic-pain treatments. The present study tested the hypothesis that contingent exposure to hindpaw mechanical stimulation might punish operant responding in CFA- and paclitaxel-treated rats, augment the magnitude and duration of pain-related behavioral depression produced by these chronic pain models, and provide a baseline of chronic pain-related behavioral depression that could be used to evaluate candidate analgesics.

Hindpaw mechanical stimulation was achieved by requiring rats to rear on a textured NOX plate developed to excite nociceptors rendered hypersensitive by a nerve-ligation model of neuropathic pain, and the stimulus associated with locomotion on this plate was found to punish locomotor activity in nerve-injured rats but not in sham controls (Boada et al., 2016). Consistent with these previous findings, rearing on the NOX plate did not punish operant responding in rats prior to treatment with CFA, paclitaxel, or their respective controls, nor did rearing on the NOX plate punish responding after control treatments. These findings suggest that mechanical stimulation associated with rearing on the NOX plate was not sufficient by itself to produce pain-related punishment of operant responding.

Responding was significantly reduced after CFA and high-dose IV paclitaxel treatment; however, three findings suggest that this reduction in operant responding was not a consequence of pain-related punishment. First, depression of operant responding was usually weaker or more transient than concurrently assessed hypersensitivity of paw-withdrawal responses to mechanical stimulation with von Frey probes. After Ipl CFA or low-dose IP paclitaxel, mechanical hypersensitivity was observed for at least three weeks as described previously, but depression of operant responding had resolved by three days after CFA and was never significant after IP paclitaxel. Second, high-dose IV paclitaxel did produce a more sustained depression of operant responding; however, this effect was small, and morphine doses shown previously to alleviate mechanical hypersensitivity in paclitaxel-treated rats (Legakis and Negus, 2018) did not increase operant responding, suggesting that the depression of operant responding was not related to pain. Lastly, the time course of operant depression after Ipl CFA was identical whether rats were required to rear on the NOX plate or not, further suggesting that the mechanical stimulation associated with rearing on the NOX plate did not augment depression produced by the CFA treatment alone. Similarly, the weak effects of low-dose IP paclitaxel on operant responding in the present study were similar to the weak and morphine-resistant effects observed previously in rats responding for food or electrical brain stimulation on a low lever with no NOX plate (Legakis et al., 2018; Legakis et al., 2019).

As one manipulation intended to increase the potential for detecting sustained pain-related behavioral depression, the present study used a high-dose IV paclitaxel treatment regimen that more closely mimics the IV doses used clinically in humans and that produces leukopenia similar to that produced by clinically effective paclitaxel dosing regimens (Hamity et al., 2017). This high-dose IV paclitaxel regimen did produce stronger effects than the low-dose IP regimen on several endpoints including 1) weight loss (sufficient to require euthanasia in one rat despite the supplemental feeding), 2) mechanical hypersensitivity after 1 and 3 weeks, and 3) significant depression of operant responding throughout the study. In contrast to results with the low-dose IP regimen in the present study or with the high-dose IV regimen in the original study (Hamity et al., 2017), the mechanical hypersensitivity was no longer significant after four weeks. Reasons for this discrepancy are not clear and may be related to methodological differences that included use of male rather than female rats, use of IV catheters rather than tail-vein injections for IV paclitaxel delivery, or repeated exposure to the operant behavioral procedure and NOX plate. Operant responding was decreased throughout the study in these rats, and this may be consistent with the previously reported effectiveness of mechanical stimulation in IV PTX-treated rats to punish locomotor behavior in the PEAP procedure (Hamity et al., 2017); however, as noted above, morphine was not effective to alleviate this effect, suggesting that the behavioral depression was not related to pain.

In addition to producing changes in behavior, inflammatory and neuropathic pain manipulations have also been reported to produce changes in endogenous opioid signaling, and two of those were examined here. First, “latent sensitization” has been described as a phenomenon in which inflammation- or neuropathy-induced mechanical hypersensitivity resolves due in part to the emergence of sustained constitutive MOR activity in the spinal dorsal horn (Corder et al., 2013; Marvizon et al., 2015). This “latent sensitization” can be revealed by the administration of naltrexone or other MOR antagonists to block antinociception associated with constitutive MOR activity and reinstate expression of mechanical hypersensitivity. In the present study, naltrexone-induced reinstatement of mechanical hypersensitivity in CFA-treated rats is consistent with latent sensitization; however, naltrexone did not reinstate depression of operant responding, suggesting that rapid recovery of operant responding did not reflect constitutive MOR activity in neural circuits mediating CFA-induced depression of operant responding. Additionally, naltrexone did not alter either mechanical sensitivity or operant responding in paclitaxel treated rats, suggesting that constitutive MOR activity did not contribute to resolution of either paclitaxel effect. Second, inflammation and neuropathy in rodents have also been reported to promote dynorphin signaling and KOR activation in the mesolimbic dopamine system, and this enhanced KOR signaling has been implicated in some signs of pain-related behavioral depression (Liu et al., 2019; Meade et al., 2020). We have not found evidence for this type of pain-related activation of KOR signaling in previous studies using both acute and chronic pain manipulations (Leitl et al., 2014a; Leitl et al., 2014b; Negus et al., 2015; Bagdas et al., 2016; Legakis et al., 2020), and the present study is consistent with our own previous findings. Thus, CFA and paclitaxel treatment produced only transient and/or weak evidence for depression of operant responding, so there was little evidence to suggest a KOR-mediated depressant effect in this study. It was possible that CFA- or paclitaxel-induced increases in dynorphin release may have occurred, but they were either insufficient to depress behavior or their impact was attenuated by compensatory KOR downregulation. To evaluate this possibility, we determined effects of the exogenous KOR agonist U69593, but dose-effect curves for U69593-induced depression of operant responding were not affected by either CFA or paclitaxel treatment. Thus, these results also do not provide evidence for either latent increases in dynorphin (which might have been additive with U69593 and shifted U69593 dose-effect curves to the left) or KOR downregulation (which might have shifted U69593 dose-effect curves to the right). Overall, our results provide no evidence for pain-related alterations in KOR signaling.

A final experiment in this study evaluated the possibility that rapid recovery of operant responding in CFA-treated rats may have reflected behavioral tolerance (Schuster et al., 1966; Sannerud and Young, 1986; Foltin, 2015), which can be defined as tolerance to drug effects due to learning. For example, rats might have learned new postures that minimized contact between the CFA-injected paw and the NOX plate to reduce exposure to mechanical stimulation associated with that contact. To test for this possibility, a group of CFA-treated rats was given access to operant responding only one week after CFA injection, and behavioral tolerance would have been indicated by delayed recovery due to delayed opportunity for learning. However, recovery in this group was identical to recovery in the rats with daily access to operant responding, suggesting that learned compensatory behaviors analogous to behavioral tolerance did not contribute to rapid recovery.

In summary, the present results provide little evidence for sustained depression of food-maintained operant responding as a sign of the “affective/motivational” dimension of pain in rats treated with CFA or paclitaxel. The rapid recovery of operant responding in CFA- and IP PTX-treated rats cannot be attributed to latent sensitization associated with constitutive MOR activity, to altered KOR signaling, or to behavioral tolerance. Only the IV PTX-treated rats showed sustained operant depression, but even here, the effect was small and resistant to treatment with the opioid analgesic morphine, suggesting that it was not related to pain. The use of mild food restriction in the present study may have increased food motivation and rendered operant responding resistant to depression by pain manipulations; however, we recently found that mild food deprivation like that used here has no significant effect of food motivation measured with a between-day progressive-ratio procedure (unpublished data). Overall, these data add further evidence to suggest that putative chronic-pain manipulations in rats may be a poor model for research on the expression, mechanisms, and treatment of the functional impairment and behavioral depression commonly observed in human chronic-pain patients.

## Data Availability Statement

The raw data supporting the conclusions of this article will be made available by the authors, without undue reservation.

## Ethics Statement

The animal study was reviewed and approved by Virginia Commonwealth University Institutional Animal Care and Use Committee.

## Author Contributions

All authors contributed to the conduct of experiments and approved the final manuscript. SN was responsible for experimental design, data analysis, and writing an initial draft of the manuscript.

## Funding

Supported by National Institutes of Health Grant P30DA033934 and F32DA047026.

## Conflict of Interest

The authors declare that the research was conducted in the absence of any commercial or financial relationships that could be construed as a potential conflict of interest.

## References

[B1] Bagdas D. Muldoon P. P. AlSharari S. Carroll F. I. Negus S. S. Damaj M. I. (2016). Expression and pharmacological modulation of visceral pain-induced conditioned place aversion in mice. Neuropharmacology 102, 236–243. 10.1016/j.neuropharm.2015.11.024 26639043 PMC5574195

[B2] Boada M. D. Martin T. J. Ririe D. G. (2016). Nerve injury induced activation of fast-conducting high threshold mechanoreceptors predicts non-reflexive pain related behavior. Neurosci. Lett. 632, 44–49. 10.1016/j.neulet.2016.08.029 27544012 PMC5310223

[B3] Brown D. C. Boston R. C. Coyne J. C. Farrar J. T. (2008). Ability of the canine brief pain inventory to detect response to treatment in dogs with osteoarthritis. J. Am. Vet. Med. Assoc. 233, 1278–1283. 10.2460/javma.233.8.1278 19180716 PMC2896492

[B4] Chaplan S. R. Bach F. W. Pogrel J. W. Chung J. M. Yaksh T. L. (1994). Quantitative assessment of tactile allodynia in the rat paw. J. Neurosci. Methods 53, 55–63. 10.1016/0165-0270(94)90144-9 7990513

[B5] Cone K. Lanpher J. Kinens A. Richard P. Couture S. Brackin R. (2018). Delta/mu opioid receptor interactions in operant conditioning assays of pain-depressed responding and drug-induced rate suppression: assessment of therapeutic index in male Sprague Dawley rats. Psychopharmacology (Berl) 235, 1609–1618. 29572653 10.1007/s00213-018-4876-x PMC5924452

[B6] Corder G. Doolen S. Donahue R. R. Winter M. K. Jutras B. L. He Y. (2013). Constitutive μ-opioid receptor activity leads to long-term endogenous analgesia and dependence. Science 341, 1394–1399. 10.1126/science.1239403 24052307 PMC4440417

[B7] Dworkin R. H. Turk D. C. Farrar J. T. Haythornthwaite J. A. Jensen M. P. Katz N. P. (2005). Core outcome measures for chronic pain clinical trials: IMMPACT recommendations. Pain 113, 9–19. 10.1016/j.pain.2004.09.012 15621359

[B8] Ewan E. E. Martin T. J. (2014). Differential suppression of intracranial self-stimulation, food-maintained operant responding, and open field activity by paw incision and spinal nerve ligation in rats. Anesth. Analg. 118, 854–862. 10.1213/ANE.0000000000000119 24651240

[B9] Foltin R. W. (2015) “Behavioral tolerance”, in Encyclopedia of psychopharmacology. Editors Stolerman I. P. Price L. H., 270–274, (Berlin, Germany: Springer).

[B10] González-Cano R. Montilla-García Á. Ruiz-Cantero M. C. Bravo-Caparrós I. Tejada M. Á. Nieto F. R. (2020). The search for translational pain outcomes to refine analgesic development: where did we come from and where are we going? Neurosci. Biobehav. Rev. 113, 238–261. 10.1016/j.neubiorev.2020.03.004 32147529

[B11] Hamity M. V. White S. R. Walder R. Y. Schmidt M. S. Brenner C. Hammond D. L. (2017). Nicotinamide riboside, a form of vitamin B3 and NAD+ precursor, relieves the nociceptive and aversive dimensions of paclitaxel-induced peripheral neuropathy in female rats. Pain 158, 962–972. 10.1097/j.pain.0000000000000862 28346814

[B12] Hodos W. (1961). Progressive ratio as a measure of reward strength. Science 134, 943–944. 10.1126/science.134.3483.943 13714876

[B13] Jensen M. P. Chodroff M. J. Dworkin R. H. (2007). The impact of neuropathic pain on health-related quality of life: review and implications. Neurol. Now. 68, 1178–1182. 10.1212/01.wnl.0000259085.61898.9e 17420400

[B14] LaBuda C. J. Fuchs P. N. (2000a). A behavioral test paradigm to measure the aversive quality of inflammatory and neuropathic pain in rats. Exp. Neurol. 163, 490–494. 10.1006/exnr.2000.7395 10833324

[B15] LaBuda C. J. Fuchs P. N. (2000b). Morphine and gabapentin decrease mechanical hyperalgesia and escape/avoidance behavior in a rat model of neuropathic pain. Neurosci. Lett. 290, 137–140. 10.1016/s0304-3940(00)01340-9 10936696

[B16] Le Bars D. Hansson P. T. Plaghki L. (2010) Current animal tests and models of pain, in Pharmacology of pain. Editors Beaulieu P Lussier D Porreca F Dickenson A. H. (Seattle, Washington: IASP Press), 475–504.

[B17] Legakis L. P. Bigbee J. W. Negus S. S. (2018). Lack of paclitaxel effects on intracranial self-stimulation in male and female rats: comparison to mechanical sensitivity. Behav. Pharmacol. 29, 290–298. 10.1097/FBP.0000000000000378 29369054 PMC5854530

[B18] Legakis L. P. Diester C. M. Townsend E. A. Karim-Nejad L. Negus S. S. (2019). Comparison of chemotherapy effects on mechanical sensitivity and food-maintained operant responding in male and female rats. Behav. Pharmacol. 31 477–490. 10.1097/FBP.0000000000000527 PMC7673225 31833969

[B19] Legakis L. P. Karim-Nejad L. Negus S. S. (2020). Effects of repeated treatment with monoamine-transporter-inhibitor antidepressants on pain-related depression of intracranial self-stimulation in rats. Psychopharmacology (Berl) 237, 2201–2212. 10.1007/s00213-020-05530-y 32382785 PMC7308219

[B20] Legakis L. P. Negus S. S. (2018). Repeated morphine produces sensitization to reward and tolerance to antiallodynia in male and female rats with chemotherapy-induced neuropathy. J. Pharmacol. Exp. Therapeut. 365, 9–19. 10.1124/jpet.117.246215 PMC5830638 29363579

[B21] Leitl M. D. Onvani S. Bowers M. S. Cheng K. Rice K. C. Carlezon W. A. (2014a). Pain-related depression of the mesolimbic dopamine system in rats: expression, blockade by analgesics, and role of endogenous κ-opioids. Neuropsychopharmacology 39, 614–624. 10.1038/npp.2013.236 24008352 PMC3895239

[B22] Leitl M. D. Potter D. N. Cheng K. Rice K. C. Carlezon W. A. Negus S. S. (2014b). Sustained pain-related depression of behavior: effects of intraplantar formalin and complete freund’s adjuvant on intracranial self-stimulation (ICSS) and endogenous kappa opioid biomarkers in rats. Mol. Pain 10, 62 10.1186/1744-8069-10-62 25245060 PMC4180532

[B23] Liu S. S. Pickens S. Burma N. E. Ibarra-Lecue I. Yang H. Xue L. (2019). Kappa opioid receptors drive a tonic aversive component of chronic pain. J. Neurosci. 39, 4162–4178. 10.1523/JNEUROSCI.0274-19.2019 30862664 PMC6529867

[B24] Marvizon J. C. Walwyn W. Minasyan A. Chen W. Taylor B. K. (2015). Latent sensitization: a model for stress-sensitive chronic pain. Curr. Protoc. Neurosci. 71, 9–14. 10.1002/0471142301.ns0950s71 25829356 PMC4532319

[B25] Meade J. A. Alkhlaif Y. Contreras K. M. Obeng S. Toma W. Sim-Selley L. J. (2020). Kappa opioid receptors mediate an initial aversive component of paclitaxel-induced neuropathy. Psychopharmacology (Berl) 237, 2777–2793. 10.1007/s00213-020-05572-2 32529265

[B26] Miller L. L. Picker M. J. Schmidt K. T. Dykstra L. A. (2011). Effects of morphine on pain-elicited and pain-suppressed behavior in CB1 knockout and wildtype mice. Psychopharmacology (Berl) 215, 455–465. 10.1007/s00213-011-2232-5 21373789 PMC3160632

[B27] National Research Council (2011). Guide for the care and use of laboratory animals. Washington DC: National Academies Press

[B28] Negus S. S. (2019). Core outcome measures in preclinical assessment of candidate analgesics. Pharmacol. Rev. 71, 225–266. 10.1124/pr.118.017210 30898855 PMC6448246

[B29] Negus S. S. (2013). Expression and treatment of pain-related behavioral depression. Lab Anim. (N.Y.) 42, 292–300. 10.1038/laban.255 PMC5425249 23877610

[B30] Negus S. S. Neddenriep B. Altarifi A. A. Carroll F. I. Leitl M. D. Miller L. L. (2015). Effects of ketoprofen, morphine, and kappa opioids on pain-related depression of nesting in mice. Pain 156, 1153–1160. 10.1097/j.pain.0000000000000171 25827812 PMC4766843

[B31] Okun A. McKinzie D. L. Witkin J. M. Remeniuk B. Husein O. Gleason S. D. (2016). Hedonic and motivational responses to food reward are unchanged in rats with neuropathic pain. Pain 157, 2731–2738. 10.1097/j.pain.0000000000000695 27548047 PMC5108682

[B32] Polomano R. C. Mannes A. J. Clark U. S. Bennett G. J. (2001). A painful peripheral neuropathy in the rat produced by the chemotherapeutic drug, paclitaxel. Pain 94, 293–304. 10.1016/s0304-3959(01)00363-3 11731066

[B33] Richardson N. R. Roberts D. C. (1996). Progressive ratio schedules in drug self-administration studies in rats: a method to evaluate reinforcing efficacy. J. Neurosci. Methods 66, 1–11. 10.1016/0165-0270(95)00153-0 8794935

[B34] Sannerud C. A. Young A. M. (1986). Modification of morphine tolerance by behavioral variables. J. Pharmacol. Exp. Therapeut. 237, 75–81. 3958974

[B35] Schuster C. R. Dockens W. S. Woods J. H. (1966). Behavioral variables affecting the development of amphetamine tolerance. Psychopharmacologia 9, 170–182. 10.1007/BF00404721 5983915

[B36] Stein C. Millan M. J. Herz A. (1988). Unilateral inflammation of the hindpaw in rats as a model of prolonged noxious stimulation: alterations in behavior and nociceptive thresholds. Pharmacol. Biochem. Behav. 31, 445–451. 10.1016/0091-3057(88)90372-3 3244721

[B37] Stevenson G. W. Bilsky E. J. Negus S. S. (2006). Targeting pain-suppressed behaviors in preclinical assays of pain and analgesia: effects of morphine on acetic acid-suppressed feeding in C57BL/6J mice. J. Pain 7, 408–416. 10.1016/j.jpain.2006.01.447 16750797

[B38] Stevenson G. W. Cormier J. Mercer H. Adams C. Dunbar C. Negus S. S. (2009). Targeting pain-depressed behaviors in preclinical assays of pain and analgesia: drug effects on acetic acid-depressed locomotor activity in ICR mice. Life Sci. 85, 309–315. 10.1016/j.lfs.2009.06.006 19559034 PMC2761814

[B39] Tappe-Theodor A. King T. Morgan M. M. (2019). Pros and cons of clinically relevant methods to assess pain in rodents. Neurosci. Biobehav. Rev. 100, 335–343. 10.1016/j.neubiorev.2019.03.009 30885811 PMC6528820

[B40] Townsend E. A. Beloate L. N. Huskinson S. L. Roma P. G. Freeman K. B. (2015). Corn oil, but not cocaine, is a more effective reinforcer in obese than in lean Zucker rats. Physiol. Behav. 143, 136–141. 10.1016/j.physbeh.2015.03.002 25744935 PMC4408761

[B41] Townsend E. A. Negus S. S. Caine S. B. Thomsen M. Banks M. L. (2019). Sex differences in opioid reinforcement under a fentanyl vs. food choice procedure in rats. Neuropsychopharmacology 44, 2022–2029. 10.1038/s41386-019-0356-1 30818323 PMC6898628

[B42] Warner E. Krivitsky R. Cone K. Atherton P. Pitre T. Lanpher J. (2015). Evaluation of a postoperative pain-like state on motivated behavior in rats: effects of plantar incision on progressive-ratio food-maintained responding. Drug Dev. Res. 76, 432–441. 10.1002/ddr.21284 26494422 PMC4715615

